# Draft Genome Sequence of an Endophytic Fungus, *Gaeumannomyces* sp. Strain JS-464, Isolated from a Reed Plant, *Phragmites communis*

**DOI:** 10.1128/genomeA.00734-17

**Published:** 2017-08-03

**Authors:** Jung A. Kim, Jongbum Jeon, Ki-Tae Kim, Gobong Choi, Sook-Young Park, Hyun-Jung Lee, Sang-Hee Shim, Yong-Hwan Lee, Soonok Kim

**Affiliations:** a Genetic Resources Assessment Division, National Institute of Biological Resources, Incheon, South Korea; b Department of Agricultural Biotechnology, Interdisciplinary Program in Agricultural Genomics, Center for Fungal Genetic Resources, and Center for Fungal Pathogenesis, Seoul National University, Seoul, South Korea; c Department of Plant Medicine, Sunchon National University, Suncheon, South Korea; d College of Pharmacy and Innovative Drug Center, Duksung Women’s University, Seoul, South Korea

## Abstract

An endophytic fungus, *Gaeumannomyces* sp. strain JS-464, is capable of producing a number of secondary metabolites which showed significant nitric oxide reduction activity. The draft genome assembly has a size of 53,151,282 bp, with a G+C content of 53.11% consisting of 80 scaffolds with an *N*_50_ of 7.46 Mbp.

## GENOME ANNOUNCEMENT

Endophytes are microorganisms that live within plant tissues without causing apparent harm to the host (1–3). They are known to affect plant growth and to protect the host plant from biotic and abiotic stresses from ambient environments (4). In addition, endophytes are known to produce a wide range of bioactive secondary metabolites exhibiting antibacterial, antifungal, antiviral, insecticidal, anticancer, and anti-inflammatory activities (5).

One such endophytic fungus is *Gaeumannomyces* sp. strain JS-464, which showed anti-inflammatory activity (6). This strain produced at least nine compounds, including three new ones that are two new C-glycosylated diakylresorcinol derivatives and a new glycosylated anthraquinone, which showed anti-inflammatory activity in lipopolysaccharide (LPS)-induced BV2 microglial cells (6). The synthesis of akyl resorcinol and anthraquinone requires a number of genes that are involved in biosynthesis pathways, including the polyketide pathway or the chorismate/*o*-succinylbenzoic acid pathway (7). To understand the biosynthetic details at the genome level, we have sequenced the genome of this potential bioactive fungus.

The strain *Gaeumannomyces* sp. JS-464 was isolated from the rhizome of a reed plant, *Phragmites communis*, which was collected from a swamp area of Suncheon Bay, South Korea. Genomic DNA was extracted from mycelia grown in potato dextrose broth for 2 days at 23°C, 120 rpm using the DNeasy plant minikit (Qiagen, Valencia, CA, USA). A total of 24.99 Gb of genome sequences were generated using the HiSeq 2000 (Illumina) and PacBio RSII platforms at Theragen Etex Bio Institute (Suwon, South Korea), representing 454.47-fold coverage. These sequences were assembled into 80 scaffolds (106 contigs), with an *N*_50_ of 7.46 Mb, using the mixed pipeline of FALCON, an assembler of PacBio reads, and SOAP*denovo* version 2, an assembler of Illumina reads (8). The respective assembled reads were merged with HaploMerger2 (9). The total length of the assembled genome was 53,151,282 bp, with a G+C content of 53.11%.

Subsequently, based on the AUGUSTUS program (10), gene prediction analysis was performed and yielded a total of 13,579 protein-coding genes. Using the previously developed gene family pipelines (11–16), 1,450 genes encoding secretory proteins, 453 transcription factor genes, 131 cytochrome P450 genes, 73 genes encoding plant cell wall-degrading enzymes, 13 genes encoding laccases, and 31 genes encoding peroxidases were predicted. In addition, 20 polyketide synthase and 20 nonribosomal peptide synthetase genes were identified by the antiSMASH software (17, 18). The sequences will contribute to the biosynthetic pathway of these newly found compounds and also serve to foster research on the biology of endophytic fungi.

### Accession number(s).

The draft genome sequence of *Gaeumannomyces* sp. JS-464 has been deposited at DDBJ/ENA/GenBank under accession no. NGZR00000000. The version described in this article is the first version, NGZR01000000.
